# Slowing Down to Accelerate: The Innovation of the Fundamentals of Integrated Care Governance

**DOI:** 10.5334/ijic.6548

**Published:** 2022-03-23

**Authors:** Mirella Minkman

**Affiliations:** 1 CEO of Vilans, National Center of Excellence in long term care and professor Innovation of organisation and governance of Integrated Care at Tilburg University/TIAS, The Netherlands

**Keywords:** integrated care governance, leadership, supervision, accountability

The world is in a hurry, but change is slow. Agendas are fully booked, labour markets are tense, and we seem already (too) late to alter the course of climate change. We have faced a crisis situation which has led us towards working on more ‘pandemic preparedness’. In many countries, evaluation programs try to draw lessons from the pandemic and its impact, yet at the same time try to speed up research activities, the provision of expert advice, and policy making in an attempt to reduce health damage and stabilize tensions in societies through rapid decision-making. However, these decisions themselves are eventually followed up by deep discussions about what the ‘right’ directions should be. These directions are deeply debated given how people’s perspectives for ‘what is right’ differ significantly.

As we have learned in dealing with the pandemic, solutions are not simply health-based, but must be addressed from a wider spectrum of responses to deal with the ‘wicked problem’ [1]. Integrated care and services are an important component for addressing such complex problems since multiple stakeholders, independent organisations, (conflicting) regulations and non-aligned values all play a role in making a collective response harder.

Policymakers, practitioners and researchers around the globe have for many years tried to ‘solve’ fragmentation and increase coherence in approaches that support better health and healthcare. For example, caring communities, citizens as partners, and intensifying prevention and primary care-based services are frequently mentioned as ingredients for future health systems.

Countries should work on overarching solutions that try to keep a broad perspective, as piecemeal reforms addressing only one aspect of the system at a time have been proven to fall short of the sustainable change necessary to address the complex problems we face today. The recent policy papers in IJIC about the last decade of integrated care in 19 countries including Belgium, Italy, the UK, Switzerland, Canada, the USA and so on, describe examples of these long term efforts. Yet, these policy experiences are often characterized by temporary impulses for ‘quick fixes’ rather than addressing broad spectrum interventions on mixed levels (local, regional, national) and, potentially as a result, in general have achieved mixed results [2,3,4,5,6]. Schroeder and Cutler recently highlighted the complexity of financial reforms needed to incentivize integrated care [7]. It is not only the healthcare system that is fragmentated, also other system issues like legislation or financial mechanisms are fragmented in itself. This increases the complexity for alignment and transformation even more.

## So how to move on?

In my editorial in 2017 I wrote about the way forward to realize more alignment between three fundamental components of integrated care 1; vision driven transformation (from a holistic perspective, not providing integrated ‘supply driven’ offers but creating integrated answers for people), 2; the organization of aligned (digital) services and 3; the innovation of governance that fits integrated care on multiple levels and scales like local, regional and national [8]. Alignment between these core components represents three major assignments that build the underlying cement for integrated person centered care. We have learned a lot about these fundamental components in recent years. For example, the importance of building relationships in alliances and networks and the underlying norms and values that matter [9]. We have learned that having an understanding and awareness of the underlying values of people gives insights for relationship building. Values become visible in behavior and choices of people and play a role in building trust, the willingness to collaborate and the braveness to let go silod ways of working to get things really done [10].

Furthermore, we know that making intelligent choices about the re-organisation of scarce resources takes courage, a rethinking of scale, and a reframing of the organisation of health- and social care at every level. It raises new horizons in what can be done with the community and what can be done by communities themselves. The coordination of expertise and resources (especially when there is a shortage of staff) can have a big impact on service experience and the organisation of integrated care. For example, in the Netherlands, intensive discussions are ongoing about the decision of the Minister of Health to concentrate child heart surgery in two academic centers instead of the current four. From an efficiency, scale and capacity perspective this is a logical decision, however families and carers emphasize the loss in quality of (family) life and person centered care.

Besides rethinking and organizing care systems at scales suitable to the country context [11], the importance and availability of data also increases, but data is not yet knowledge. How can we transform this data into usable knowledge for improving integrated care? What kind of data do we need most, and what is just ‘nice to have’? Knowledge is crucial to moving forward, but it is not the same as wisdom which is eventually needed for effective (policy) decision-making. Defining what data we need to make the best decisions (e.g. quality, costs, geographic data, service level, client experience, other) is linked to our underlying values and the paradigm through which we understand ‘good practices’ to look like.

A recent analysis of analytical perspectives of published papers in integrated care was described by Kemenade et. al [12]. It illustrates that the empirical paradigm and the reference paradigm are still dominant, paradigms in which measurements by indicators or results described in models and pathways are dominant. An interesting question is if other paradigms can be (more) supportive to understanding the complex, multilayered, ‘wicked issues’ that integrated care seek to address. For example, studies from a more reflective paradigm merely *discuss* the results achieved by integrated care, not measures in how to achieve it. Overall, the multi-layered wicked problem context of integrated care implies the need for intensive learning approaches and learning loops within and between organisations. This makes the development of effective integrated care in societies a long-term achievement which should be based on strong fundamentals. Essentially, we can only accelerate in the long run if we slow down to build these essential fundamentals.

## Governance fundamentals

These fundamentals bring us back towards the role of the governance of integrated care. The assignment for the next decade is to transform governance modalities -also on local and regional levels -that suit integrated care to contribute to (interdisciplinary) wicked problems in societies. Governance modalities are often not well designed to suit this purpose, but are single perspective, professional or organizational driven and often risk-avoiding instead of future and transformation driven. Governance components that need those transformation by re-design are leadership, supervision, and accountability procedures. Supervision includes both internal supervision (for instance by supervisory boards) and external supervision (for instance by Inspectorates/legislators). Supervisors traditionally have a responsibility and focus on the entity of the healthcare organisation. When supervisors focus (only) on reducing risks, maintaining budgets and optimizing their own organisation instead of looking at the contribution they can offer to address a broader wicked problem as defined by Head [1], it is easy for leaders to avoid real innovation which is needed for integrated care. Also, care and support is more and more delivered by inter-organisational networks, which asks for another modality than single-organisational boards. In the Netherlands, our National Governance Code for health care organisations was updated this year [14]. For the first time, it now mentions that the responsibility for good governance does not end within the organization itself, but also includes the collaborative networks in which the organisation participates. The governance of these networks can be diverse and is not described in this code, which leaves room for multiple modalities and also discussion (and research) about what works best when. Further, external supervisors like inspectors or regulators still focus merely on the professional delivery or quality and costs of the organisation [15]. On top of this, both types of supervision are often not aligned or there is even no communication at all between supervisory bodies.

Leadership approaches, as another component of governance asks for different competences and personalities in integrated care settings. Training in these competences for leaders are upcoming, but are not widely invested in [13]. Competences like adaptiveness and being able to bridge different views and non-aligned interests are important. Therefore it is even more important that not only leadership itself, but also accountability procedures and supervision of the boards/leaders incentivize leaders in their complex task. This task consists of two parts: driving forward innovation and maintaining the daily business which make boardroom dynamics challenging and heavy tasks.

## The importance of governance

In many countries, we are not there yet. Besides innovating modalities and approaches for governance, the question is who is involved. Citizens, clients, or patients and their families do have an important voice which needs to be heard, and they have representative bodies, which need consideration to include in these networks. However, often, they don’t play a significant role in network governance, if they are included at all. Single health care organisations often have a legal requirement to institute a client board or council, as well as having employee representation in the governance structures. However, with integrated care new questions emerge about how and where to organize these boards or councils. Or better, if these ways of working do fit integrated services. ***Table 1*** summarizes the challenges in integrated care governance.

**Table 1:** Transformation of integrated care governance.


GOVERNANCE COMPONENT	TRANSFORMATIONAL DIRECTION

*Organisational performance* as **leadership** challenge	Adaptiveness to and participating in *context fitting networks* for wicked problems as **leadership** challenge

(Internal) **supervisory boards** focusing on risk reduction, quality, costs and reputation	**Supervisory boards** focusing on *shared responsibilities* in alliances for societal wicked problems

**External supervision** focusing on professions or organisations	**External supervision** on interdisciplinary work and inter-organisational networks

**Involvement of employees** in governance structures	Involvement of **inter-organisational working employees** in network governance

**Client or citizen** involvement in client boards/councils	**Governance modalities** that incorporate client or citizens voices in inter-organisational networks

**Accountability** towards payers and regulators	**Accountability** towards society (sustainability, environment, use of resources)


Without the innovation of these fundamental components of integrated care governance, there is a risk of sub-optimalisation. Therefore, to really accelerate in this rushed timeframe, slowing down to work on integrated care governance could be crucial for the future. Politicians may not like this message. However, there is no need to wait for working on integrated care governance. Innovation of governance (unfortunately) takes time because it has to break down comfortable ways of working. Quick wins may look attractive, but investing in adaptive leadership, learning organisations and inter-organizational networks with suitable modalities of accountability and supervision may ask for slowing down before accelerating. Knowledge and research about these integrated care governance modalities may bring the wisdom that is eventually needed.
